# Analysis of Heavy Metal Contamination of Agricultural Soils and Related Effect on Population Health—A Case Study for East River Basin in China

**DOI:** 10.3390/ijerph17061996

**Published:** 2020-03-18

**Authors:** Liping He, Wei Hu, Xiaofeng Wang, Yu Liu, Yan Jiang, Yanbin Meng, Qipeng Xiao, Xinxin Guo, Yanfeng Zhou, Yongyi Bi, Yuanan Lu

**Affiliations:** 1 School of Public Health, Xiangnan University, Chenzhou 423000, China; wangxf20161123@126.com (X.W.); ly8923028@126.com (Y.L.); jyan89@126.com (Y.J.); myanbin@163.com (Y.M.); 2olegend.gs@163.com (Q.X.); guoxinxin@126.com (X.G.); yanfengzhou_h@163.com (Y.Z.); 2 Department of Epidemiology and Health Statistics, Guangdong Medical University, Dongguan 523808, China; huw1991@hotmail.com; 3 School of Public Health, Wuhan University, Wuhan 430072, China; yongyib@aliyun.com; 4 Department of Public Health Sciences, University of Hawaii at Mānoa, 1960 East-West Road, Honolulu, HI 96822, USA

**Keywords:** heavy metals, agricultural soil pollution, blood lead, East River basin, Chenzhou

## Abstract

To understand the heavy metal pollution in agricultural soils along the East River basin and assess the pollution related health effect to local residents, interviews and archived data were obtained to identify the study sites affected by polluted tailing. Soil samples were collected and tested for heavy metal content and the Comprehensive Pollution Index (CPI). The degree of pollution of agricultural soils in the area was assessed using GIS-based Spatial distribution map of heavy metals and the trend of soil heavy metal risk. Two villages (Matian and Zhudui) near the East River were included in this study for health effect assessment. A total of 193 residents aged 15 or above from each village were tested for the present status of chronic diseases. Convenient sampling method was used to collect blood samples from 78 residents for heavy metal concentration. The contents of Pb, Cd, As, Zn, and Cu in the agricultural soils were all over the standards with a moderate to severe CPI. Among these metals, Cd was the highest followed by Pb, and Cu was the lowest. The contents of Pb, Cd, As, and Zn tend to be higher in soils closer to the river. The prevalence of chronic diseases was over 30%, which is significantly higher than the report from the national central region (23.15%). The average blood lead level (BLL) among children under 14 years is 7.42 μg/dL. Although the adults in Matian village had a significantly higher BLL (χ^2^ = 8.70, *p* = 0.03) as compared to Zhudui village, there was no significant difference for the prevalence of chronic diseases between the two villages (χ^2^ = 3.23, *p* = 0.09). The mean BLL of children and the proportion of children with BLL ≥ 10 µg/dL in this study are equivalent to the national average. The higher BLL concentration and prevalence of chronic diseases in adults might be due to their long-term exposure to heavy metal contamination environment and higher background level of heavy metals. Findings from this study will form the baseline information for local government to the development of effective approaches to control the heavy metal contamination and reduce the pollution related adverse health effect on local residents.

## 1. Introduction

A total of 990 carcinogens in four categories were published by the International Agency for Research on Cancer (IARC) including heavy metals, such as cadmium (Cd), arsenic (As), nickel (Ni), lead (Pb), cobalt (Co), mercury (Hg), beryllium (Be), and chromium (Cr), and their related complexes [1]. In addition to carcinogenic effect, exposure to heavy metals can also cause non-carcinogenic health impacts such as destruction of neurological, immune, respiratory, and reproductive function [2,3,4,5].

Geographical information system (GIS) has been increasingly used to monitor and simulate the migration of heavy metals in the environment and their effects on population health. In addition, GIS was also used for the exploration and identification of heavy metal pollution sources [4,6], the assessment of the risk of heavy metal pollution [7], and the studies on heavy metal pollution-mediated both carcinogenic [8,9] and non-carcinogenic effects [10,11,12,13].

Chenzhou City in Hunan Province is referred the “hometown of nonferrous metals in the world”, possessing 143 mining resources and 310 million tons of polymetallic deposits. The East River (also known as Dong River and Dalangjiang) is located in a high-tech zone in the northeastern part of Chenzhou City, with a total length of 33.84 km. It is a secondary tributary of the Xiang River and a primary tributary of the Lei River. The upper reaches of the East River are mountainous and steep terrain, while the lower reaches are flat plains. The upper region is known to contain nonferrous metal deposits. Historically, more than 70 private and individual mining companies were there on both sides of the river, resulting in the piling of beneficiation tailings and heavy metal pollution. The upper reaches are the Shizuyuan mining regions with large-scale state-owned non-ferrous metal companies conducting integrated mining, separating, and smelting of nonferrous metals. There are four tailing dams in this mining area. Yejiwei tailings dam is one of them and collapsed on 25 August 1985 due to a flood, resulting in discharging more than 2 million m^3^ of tailings. These tailings were washed along with river water for more than 10 km to the downstream Shihupu village. Although sludge and contaminated topsoil were urgently disposed immediately after the incident, however, heavy metal contamination is still detectable in the water, soil, and vegetables in the East River basin, including lead (Pb), cadmium (Cd), arsenic (As), and zinc (Zn) [14,15]. In addition to remediate cleaning up and gradually standardized mining, the “Dong River Mining Environment Geological Treatment Project” was officially initiated in 2012 and completed in 2013. Furthermore, river tailings have been cleared and transported, and the construction of irrigation and drainage projects and ecological restoration have been completed. Great changes have taken place on both sides of the River now, including the lifestyles and dietary paths of local residents.

This paper reviewed the literature from 1985 to the present and examined the content of heavy metals in agricultural soils in the study area. This study describes the degree and distribution of agricultural soil pollution in the vicinity of the East River area affected by tailings. Participant residents were examined for their health conditions such as blood lead and chronic disease prevalence to understand the health risks of heavy metal pollution in the area. Our new findings will form the scientific basis essential for further cleaning up of the contaminant heavy metals from the region and promote public health of the local residents.

## 2. Materials and Methods

### 2.1. Study Region and Soil Sampling Sites

From 2012 to 2013, members of the study team conducted a heavy metal contamination related inquiry through historical data collection, personal interviews, and on-site inspections. The historical data of the East River basin was mainly obtained by reviewing the archives from the Suxian District Bureau, while on-site observation focused on topography, the distribution of villages and vegetable fields near the East River in Suxian District. Personal interviews were conducted to collect data concerning the living habits of local residents, mining industry and agriculture, changes in natural and society eco-system, specifically to the collapse and subsequent treatment of the Yejiwei tailing in 1985. The interviewees included local government officials and authorities, officials of the environmental protection administrative department, local mining or mineral processing plant managers and staff, and long-term residents. Finally, inductive analysis of collected data was carried out to determine the study region and sampling sites.

### 2.2. Sources of Heavy Metal Content in Agricultural Soils

#### 2.2.1. Literature on the Collapsed Tailings and Heavy Metal Pollution in Affected Region

The literature search was restricted to papers published in Chinese and English after 1985 on heavy metal contamination to the study region. The search strategy was defined according to PECO principles: P (Project) represents the study subject (or region), including the East River, river of east, Chenzhou, southern China, southern Hunan; E (Exposure) represents the study exposure factors, including heavy metals Pb, Cd, As, Zn, and Cu; C (Comparison/control) represents the study control factors or the standard reference value.

Two reviewers independently evaluated the search results. With a third independent reviewer, they discussed the quality of the research presented in each published paper and the relevance of the work to quantitation of heavy metal contamination in soils in the study region. Literatures on the collapsed tailings and heavy metal pollution in affected regions are summarized in Table 1.

#### 2.2.2. Soil Sample Collection and Heavy Metal Quantification

Selection of sampling site and sample collection were carried out according to the People’s Republic of China Environmental Protection Industry Standards (HJ/T 166-2004) [16]. Five sampling sites were determined for Matian village where the East River water was used for irrigation, and three sampling sites were set up for Zhudui village in the east bank of the river, where the Shan river water used for irrigation. A handheld GPS device was used during sampling times to record the geographical coordinates. According to the terrain type, different sampling methods including plum-shape, serpentine, or diagonal methods were used. Five soil samples were collected at 10–20 cm deep from the surface of every sampling site. Each sample of 200–500 g of soil was collected, mixed, packaged in polyethylene bags, coded, and recorded before transporting to the laboratory. Soil samples were allowed to dry by placing them in a well-ventilated laboratory hood. After removing gravel, plant materials, and other debris with a 2-cm sieve, 100 g soil sample was ground down with a wooden stick and passed through a nylon sieve of 100-mesh. A ZEENit 700 atomic absorption spectrophotometer equipped with an MPE60 automatic sampler (Jena, Germany) was used to detect heavy metals in soil samples. Quantitation of heavy metals in soils was carried out in the Guiyang Disease Control and Prevention Center (DCPC) according to the methods in the People’s Republic of China Environmental Protection Industry Standards (HJ803-2016) [17].

### 2.3. Evaluation Parameters and Calculation Methods for Soil Heavy Metal Pollution

#### 2.3.1. Evaluation Method for Single Pollution Index

Equation (1) was used to calculate the single metal pollution index of each soil sample:

(1) $$P_{i}=\frac{C_{i}}{S_{i}}$$

In the formula, *P_i_* is the individual pollution indexes of different heavy metals in the soil compared with different standards. *Ci* is the actual content of each heavy metal element in the soil. *Si* is the standard reference values of each heavy metal element. Newly published [18] “Soil Environmental Quality in Agricultural Soils” in 2018 were used as the risk screening values of lead, cadmium, arsenic, copper, and zinc and risk control values for lead, cadmium, and arsenic, respectively, in this study (Table 2).

#### 2.3.2. Soil Comprehensive Pollution Index Model and Evaluation Method

In this paper, the comprehensive pollution status of heavy metals in soil is evaluated by the comprehensive pollution index method (Nemerow Index method) [19] widely used in environmental assessment. The comprehensive pollution index method incorporates the weights of various heavy metal elements into the model in addition of considering the effects of different heavy metal elements on the soil. Based on the calculation of the individual pollution index of each heavy metal, the comprehensive pollution index is obtained.

Nemerow index evaluation model:

(2) $$P=\sqrt{\frac{{\left({\bar{P}}_{i}\right)}^{2}+{\left(P_{max}\right)}^{2}}{2}}$$

where *P* is the comprehensive pollution index of the soil in the study site. $P_{max}$ is the single-factor pollution index with the highest value among pollutants. ${\bar{P}}_{i}$ is the average single-factor pollution index of the pollutants, and it is calculated as follows:

(3) $${\bar{P}}_{i}=\frac{1}{n}\sum_{i=1}^{n}P_{i}$$

In the equation, *n* is the number of pollutants evaluated, $P_{i}$ is the single-factor pollution index of various heavy metals in soil samples. $C_{i}$ is the measured concentration of a heavy metal in soil samples. $S_{i}$ is the standard reference value of a particular pollutant. The standard reference value adopted in this paper was the second standard values in the National Environmental Quality Standard for Soil [18]. According to the National Environmental Quality the second level, which states that Pb is under 50 mg/kg, Cd is under 0.30 mg/kg, As is under 25 mg/kg, Cu is under 200 mg/kg, Zn in under 250 mg/kg, and Cr is under 250 mg/kg. The comprehensive pollution index is divided into five grades: *P* ≤ 0.7 is clean (safe category), 0.7 < *P* ≤ 1 is the warning line, 1 < *P* ≤ 2 is mild pollution, 2 < *P* ≤ 3 is moderate pollution, and *P* > 3 is severe pollution.

### 2.4. *Health Surveys for Residents in the Study Site*

The study was approved by the Institutional Review Board of Xiangnan University (No. 2012-29). All subject information was kept confidentially.

#### 2.4.1. Questionnaire Survey

The household survey was completed in August 2013 in the research area and subjects from Matian and Zhudui two villages were selected according to screening criteria using a voluntary principle.

The criteria for the respondent inclusion were 15 years old or older who had lived there more than 5 years, and the exclusion criteria included those who were unable to answer the questionnaire due to mental or physical reasons. According to the geographical location and administrative district, 210 households with at least one member/household meeting the conditions of the study were randomly targeted for the survey, but only 193 residents completed the questionnaire properly.

The survey was conducted in the daytime by the trained preventive medicine students of Xiangnan University through face-to-face interviews. The questionnaire was designed to obtain the basic information according to the chronic disease expression index in the China Health and Family Planning Statistical Yearbook [20], and the two-week prevalence rate and the chronic disease prevalence rate were included in the questionnaire. Prior to the formal investigation, a pre-survey with 20 residents in the study area were conducted and the questionnaire was improved through the pre-survey accordingly. To improve the response rate, the local village committee and the village clinics helped to conduct mobilization and notifications from house to house before the investigation. In addition, some publicity materials and gifts were provided to respondents during the survey.

#### 2.4.2. Blood Sample Collection and Blood Lead Detection

A total of 78 blood samples were conveniently collected from local residents of the two villages (Matian and Zhudui) and each sample was drawn by certified nurses after participants signed a study consent form. This study received IRB approval from the Human Subjects Review Committee of Xiangnan University. These blood samples were stored in a refrigerator at 4 °C and analyzed within 7 days. Heavy metals in the blood were detected according to the method reported by Yun et al. in 2009 [21].

### 2.5. Geographical Analysis

#### 2.5.1. Establishment of Geographical Information Database

The ArcGIS 10.2 software was used to construct a geographical information database in the following steps: (1) acquisition of geographical information: Google Earth was used to acquire the electronic location of the East River basin and surrounding areas to generate a study map. The boundary information of the surrounding villages along the East River basin and geographical location of the villages were obtained from the Suxian district governmental network and the official website of the Chenzhou urban planning bureau. (2) Data import: the study base map was directly imported as raster data, and the information stored in the Excel spreadsheets was entered into the geographical information database through File → Add Data → Add XY Data in ArcMap. (3) Definition of coordinate system: Google Earth with GCS_WGS_1984 geographical coordinate system was used to mark the study sites accurately, the geographical coordinate system of the imported base map documents and the document containing geographical information collected by Google Earth were defined as GCS_WGS_1984. (4) Definition of projection: to reduce error due to deformation during the projection process, the vector data from Excel spreadsheets was converted from GCS_WGS_1984 to Beijing_1954_3_Degree_GK_CM_111E projection coordinate system. (5) Vectorization: vectorization of required elements from the base map was carried out through the Editor tool on Arcscan and ArcMap to generate the corresponding sharp file document.

#### 2.5.2. Data Visualization and Geographical Information Analysis

In ArcMap, vectorized data was superimposed, combined, and underwent size adjustment before legends, a compass, and scale were added. The corresponding thematic map was then generated for spatial descriptive analysis and trends analysis.

### 2.6. Data Entry and Statistical Analysis

Questionnaire data was entered using EpiData, while data from the literature and soil samples were entered using Excel. Statistical analysis was carried out using the R i386 3.3.2 software for Chi-squared test, with a test level of two-tailed *α* = 0.05.

## 3. Results

### 3.1. *Generality of the Study Area*

**Figure 1:**
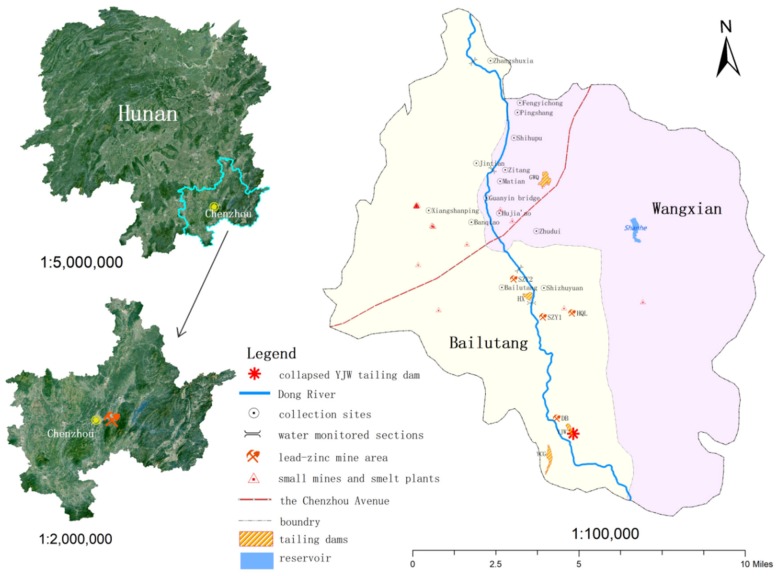
Map of the study area showing collection sites (as identified in Table 1), the lead-zinc mine area and small mines around East (Dong) River, four tailings dams, and the collapsed Yejiwei (YJW) tailings dam.

The survey results showed that the collapsed tailings sand was mainly distributed from the Shizhuyuan Village in the upper reaches of the East River to the Shihupu Village in the downstream. Therefore, the East River basin affected by the tailings sand and the tail sand of the Shizhuyuan Mine’s wild tail tailings dam were selected as the research site of this study (Figure 1). Comprehensive interviews and investigation results revealed that the study area has been basically urbanized on the west bank of the East River since 2013 with a greenway and many small parks built along the river. In the study area, only four human-made villages including Bailutang, Matian, Shizhuyuan, and Zhudui, had spaces for growing vegetables. Self-sufficiency rate of local residents’ vegetables was over 90%. In 2013, agricultural soil sampling sites were selected in addition of selecting Matian and Zhudui two villages for resident health. The study region is located in Suxian district of Chenzhou city of Hunan Province. Chenzhou is located at longitude 112°53′–113°16′ east and latitude 25°30′–26°03′ north, with an altitude of 186–500 m. The annual average temperature is 15.4–18.3 °C, annual average annual rainfall is 1250–1700 mm.

### 3.2. Analysis of Heavy Metals in Environmental Soils in East River Basin

#### 3.2.1. Literature on Heavy Metals in Agricultural Soils

As shown in Table 3, literature screening revealed 13 reports of heavy metal pollution in this region between 2002 and 2016, including five papers in English, seven papers in Chinese, and one unpublished paper from our laboratory focusing on local heavy metal contamination in soils conducted in 2013. The major pollutant metals in the soil of this region were Pb, Cd, As, Zn, and Cu.

#### 3.2.2. Concentrations of Heavy Metals in Agricultural Soils

Agricultural soil samples were collected from 14 sites of nine different villages from the study area, including Shizhuyuan, Banqiao, Jintian, Xiangshanping, Matian, Bailutang, Zhudui, Zhangshuxia, and Shihupu (Table 3). All agricultural soil samples were yellow soil with pH values ranging between 6.5 and 7.5.

As shown in Table 3, average Pb concentration among all 14 agricultural soil samples was much higher than the national standard limits (120 mg/kg). With the exception of Fengyichong, the Cd level from all samples exceeded the national standard limits (0.3 mg/kg). Arsenic concentration in 11 of the 14 sites exceeded the national standard limits (30 mg/kg). Average level of Zn detected in Zhudui, Matian, Fengyichong, and Xiangshanping four sites ranged from 11.27 to196.06 mg/kg, which is lower than national standard limits (250 mg/kg), but the other 10 sites were all higher than the standard value. The Cu concentrations in the Guanyin bridge and Shizhuyuan village samples were 135.83 and 109.24 mg/kg, respectively, which are higher than the national standard limit (100 mg/kg), but the other sites were lower than the standard limit.

#### 3.2.3. Heavy Metal Pollution of Agricultural Soils in the East River Basin

**Figure 2:**
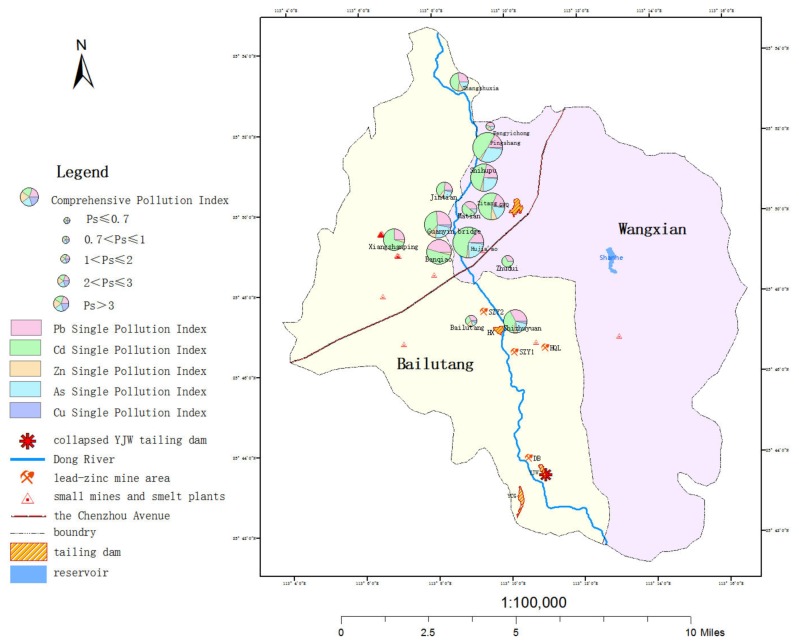
Comprehensive pollution index and single pollution index contributions of five heavy metals in agricultural soils along the East (Dong) River basin.

The Nemerow Comprehensive Pollution Index was used to evaluate the degree of heavy metal pollution in the agricultural soil in the study sites along the East River basin. The results showed that in addition to the moderate pollution detected in Fengyichong and Zhudui villages, the other 12 sites showed more severe pollution (Table 4). GIS was used for spatial descriptions of these results, which showed that the pollution index of the sampling sites on the eastern bank of the river was larger than the corresponding west coast test sites (Figure 2). At the same time, the proportions of Cd, Pb, and As were higher than the other metals in the comprehensive pollution index, and Cd had the highest contribution.

### 3.3. Health Survey

#### 3.3.1. Blood Lead Content of the Survey Population

Seventy-eight blood samples were collected from the residents of Zhudui and Matian with the age range between 3 and 59 years old. Of which, children aged ≤14 years old accounted for 32.05% and the remaining subjects (67.95%) were adults aged 15–59. Basing on the China’s standard of maximum limit of ≥ 10 µg/dL Pb in blood, 45.3% of adult bloods exceeded the Pb limit. The average blood Pb concentration for children under 14 years was 7.42 μg/dL, and 16.0% of the children with blood lead level exceeded the limit. Blood Pb levels of 0, 5, 10, and 20 µg/dL– were used to divide the population into four groups. There was no statistically significant difference in BBL among the children ≤ 14 years between the two villages (χ^2^ = 3.16, *p* = 0.21). However, the concentrations of Pb in adult blood samples collected from Matian village was significantly higher than that of the Zhudui village (χ^2^ = 8.70, *p* = 0.03) (Table 5).

#### 3.3.2. Prevalence of Chronic Diseases in the Survey Population

Among the 193 participants in this questionnaire survey, the average age of 49.44 ± 17.16 years old with a range between 15 and 81. The prevalence of chronic disease among the participants reached 30.1%, which is significantly higher than the disease prevalence in rural residents of the same age in the central region of China (23.15%) (binomial test, *p* = 0.02). As shown in Table 6, there was no statistically significant difference in the disease prevalence between Matian (39.0%) and Zhudui villages (26.1%) (χ^2^ = 3.23, *p* = 0.09).

## 4. Discussion

### 4.1. Analysis of Heavy Metal Pollution in Agricultural Soil in the East River Basin

The land stratigraphic structure of the studied area includes flat, hillock and hill, which are mainly composed of quaternary loose deposits, red rock, limestone, and sand shale. Red-, yellow-, and yellow-brown soils accounted for more than 70% of the soil plane distribution in Changzhou. In the studied area, the upper part of the river is steep and topography, where there were almost no residents there. In the middle and lower reaches, there were seven administrative villages and three primary schools. In this paper, all soil samples in this study were yellow soil with pH values ranging between 6.5 and 7.5. It is necessary to compare the results obtained in our study with other studies that were applied in agricultural soils from different regions, so as to evaluate heavy metals in agricultural soils reasonably [22]. A previous report indicated that the background values of Pb, Cs, As, Zn, and Cu in Hunan were 29.7, 0.126, 15.7, 94.4, 27.3 mg/kg, respectively, which are obviously higher than those reported for the entire country (27.3, 0.079, 13.6, 88.6, 25.4 mg/kg, respectively) [23]. In this study area, the mean concentrations of Pb, Cd, As, Cu, Zn (542, 5, 369, 362, and 65 mg/kg, respectively) in soil samples exceed the Chinese Risk screening values of agricultural soil (120, 0.3, 30, 250, and 100 mg/kg, respectively). These values also exceed the standard of the world guidelines recognized by World Health Organization (WHO) limit Pb, Cd, Cu, Zn (20, 0.3, 4, and 50 mg/kg) [22]. However, the detected values for Pb and Cd from this study area are lower than the concentrations measured for the soil near the Chenzhou mine. In 2009, Zhou et al. reported that the mean concentrations of Pb and Cd from agricultural soils near Chenzhou mine were 1531.76 and 8.79 mg/kg, respectively [24]. The Pb and Cd concentrations in the Qingshuitang industrial zone of Chenzhou were 504.03 and 12.43 mg/kg, respectively, while 556.38 and 8.81 mg/kg in Hengyang and Shuikoushan, respectively. A previous study showed that the mining of nonferrous metals in Hunan province resulted in Pb, Cd, and As contamination of 2.8 × 10^4^ km^2^ of agriculture land, accounting for 13% of the area of the entire province. There were more than 20 mining areas and numerous processing areas along the East River basin in addition of four large-scale tailings reservoirs with capacity exceeding 2 million m^3^, namely Gaowanqiu (GWQ), Hexi (HX, already closed), Yejiwei (YJW), and Yanchonggou (YCG) dams. The East River basin is rich in mineral deposits and many types of minerals. The data collected in this paper was from 13 studies and some sampling points were near the tailings dam. Now, after the “Dong River Mining Environment Geological Treatment Project” completion in 2013, 65 km green way and 10 parks have been built along the River. Those villages in the west bank of Dong River including BLT village, BQ village, and JT village, have basically urbanized. Since 2012, the farmland soil has not been allowed to be used for growing rice, and agricultural soil area has been reduced by half. The area has been mainly used for planting trees and grass except for some sporadic soils for vegetables. More than 90% of dietary vegetables of the residents are now produced/supplied by themselves. Additionally, tap water and bottled water are the main source of their drinking water. There were quite a few studies that investigated and analyzed heavy metal concentrations in agricultural soils and the health risks associated with heavy metal contamination in the East River basin. Therefore, five commonly reported heavy metals namely Pb, Cd, As, Zn, and Cu were selected for metal contamination analysis in this study.

By calculating the comprehensive pollution index, it exhibited a moderate to severe pollution for the area. Among these contaminated metals, Cd level is the highest, followed by Pb and As, with Cu to be the lowest. The concentrations of Pb, Cd, As, and Zn are higher as it is closer to the East River, which could be due to local residents using the East River water for farmland irrigation. The overall lower detection of the five heavy metals in Zhudui village may be due to where Shan River water instead of East River water was used for irrigation. The detection of abnormal distribution of Cu in Hujiatun and Xiangshanping villages which are far away from East River, may be due to the sewage and other discharge from the small-scale mining or a smelting plant nearby. The polluted water discharged to these mining areas may cause severe pollution of the surrounding soils. From the spatial trend of distribution between the north and the south, heavy metal pollution is largely due to upstream mining and processing activities. The movement of the East River water also plays a crucial role in the migration of heavy metals. A recent national survey has shown that more than 19% of agricultural soils in China are affected by heavy metal pollution, indicating rapid increase at a greater rate. Therefore, control and prevention of agricultural soils from heavy metal pollution have become urgently imminent. The concept of filter value and regulation value can be used to classify soil environmental quality. According to the different pollution degree of heavy metals in agricultural soil, the corresponding control measures were put forward to meet the requirements of “soil 10” for classified management of agricultural land [18].

### 4.2. Analysis of Association between Heavy Metal Pollution and Residential Health

The national mean for BLL in children was 9.29 µg/dL from 1995 to 2003 in China [25], and the lead poisoning rate was 33.8%. In 2002, the Chinese CDC [26] conducted a study in 19 cities and revealed the overall mean Pb concentrations in urban children aged 3–5 was 8.83 µg/dL and the proportion of children with BLL ≥ 10 µg/dL was 29.91%. In 2003–2007 [27], the mean BLL of children in China was 8.07 µg/dL, and the proportion of children with BLL ≥ 10 µg/dL was 23.9%. In 2008–2012 [28], the mean BLL of children in China was 6.32 µg/dL, and the proportion of children with BLL ≥ 10 µg/dL was 12.31%. These studies show a decreased trend of BLL in children with time in China. The mean BLL of children in this study is 7.42 µg/dL, and the proportion of children with BLL ≥ 10 µg/dL is 16.0%, which is equivalent to the national average. These findings are also consistent with the mean values reported from cities and counties around Chenzhou City [29]. In this study, the proportion of individuals aged 18 years and older with BLL ≥ 10 µg/dL was 45.3%, which is statistically higher than that reported in some regions in China, such as Chongqing City [30].

There was no statistically significant difference in BLL among participants less than 18 years old between Matian and Zhudui two villages. However, the BLL among the adults (≥18 years) was significantly higher in Matian than in Zhudui. This could be due to the fact that Matian is nearby abundant mineral resources and mining activities. It should be noticed that mining regulations and environmental remediation have recently been implemented in the region, which may explain the low detection of BLL in children in the village because of a shorter exposure as compared to the adults.

Although the BLL of children residing in this study is similar to other areas in China, there is a large difference when compared with the BLL of children in developed countries, such as the United States [31] and Canada [32]. Previous studies indicated that lead exposure in early children could result in permanent neurological damage and BLL of 5 µg/dL or higher is sufficient to cause changes in neurological behavior [33]. It is known that lead in environmental soil is one of the many factors affecting BLL, and others include living conditions, lifestyle habits, parental occupation of children, and lead-containing toys and paints. Therefore, multi-sectoral and multi-channel interventions and measures are necessary as early as possible.

Adverse health effects from chronic disease due to environmental pollution, with low doses of heavy metal contamination, require a long period of time to develop. Thus, our study selected chronic disease prevalence as a marker to study the effects of heavy metal pollution on population health. Our study demonstrated that there was no statistically significant difference between the regions with or without using East River water for irrigation. The chronic disease prevalence of the participants in this study was over 30.1%, which is significantly higher than the rate reported for rural children aged ≥15 years in the central region of China (23.15%) [17]. However, the prevalence detected in this study is comparable to or lower than that reported from other regions. For example, Zhang et al. reported [34] the chronic disease prevalence was 29.9% in rural villages in China in 2013. In addition to the relatively older age of the subjects (49.44 ± 17.16 years) enrolled in this study, the main reason for higher chronic disease prevalence in residents is due to higher background of heavy metals in this region. More adults work in mining and ore-dressing occupations, and the local resident diet relies on vegetables grown in the contaminated soils of the region. The intake of metals and long-term exposure to toxic levels of them can occur for humans through consumption of contaminated agricultural products, drinking water containing heavy metals and/or accidental exposure to heavy metals in the environment. The gradual accumulation within human tissues could create a chronic toxicity and susceptibility to cancer [35].

### 4.3. Conclusions

This study confirmed that the agricultural soil from the selected area was heavily polluted with main pollutants of Pd, Cd, As, Zn, and Cu. At present, there are no tailing and waste residues in river channels and riverbanks, and there is basically no out-of-standard phenomenon in river monitoring. The mean BLL of children and the proportion of children with BLL ≥ 10 µg/dL in this study are equivalent to the national average due to the regional pollution control effect. However, the study area is based on the exposed scene of sensitive land use, so the children may be exposed through soil and vegetable pathways, which might expose them to the polluted areas for a long time and cause harmful effect to their health. The higher BLL and high prevalence of chronic diseases among participant adults are likely due to long-term exposure to the environment with heavy metal contamination and higher background value of heavy mental. These new findings will provide baseline information to local governments for the development of more effective measures to deal with the heavy metal pollution of agricultural soils and improve health condition of local residents.

### 4.4. Limitations and Future Work

In this study, the number of soil samples and survey participants were relatively small. Heavy metal pollution has wide range of health effects, including acute poisoning, chronic nervous and blood system damage, and carcinogenic teratogenicity. The prevalence of chronic diseases was used as a marker in this study, but the accuracy of this marker in representing overall population health is debatable. In future studies, more sensitive epigenetic markers may be used to better describe the health effects of environmental heavy metal pollution.

Since 2011 the government has made considerable investments to the East River basin through several comprehensive environmental management projects. Preliminary work was completed in 2013. However, regular monitoring of heavy metal contamination of the local agricultural soil needs to be continued in addition of assessing related health effects. The cohort studies will be performed in children and more sensitive markers will be adopted to evaluate resident health in this region.

## Figures and Tables

**Table 1:** Literature on the collapsed tailings and heavy metal pollution in affected regions.

Source	Study Site	Sampling Points	Sample Depth (cm)	Analysis Method	Examination Markers
Liu 2005	East River basin	Shizhuyuan village, Jintian village, Guanyinqiao	0–15	ICP-MS	Sc, Co, Ni, Cu, Zn, As, Cd, Pb, Zr
Zeng 2006	Shizhuyuan mine	Shihupu village	20	SDDC	As
Zhou 2011	Shizhuyuan mine	Xiangshanping village, Matian village, Banqiao village, Shihupu village	0–20	AAS	Pb, Cd, Zn
He 2007	Shizhuyuan mine	Bailutang village, Shihupu village	0–20	AAS, AFS	Pb, Cd, As, Cu, Zn
Lan 2015	Shizhuyuan mine	Pingshang, Shihupu village, Zitang, Hujiaao	0–20	AAS, AFS	Pb, Cd, As, Cu, Zn
Lu 2011	Shihupu village	-	Not specified	AAS	Pb, Cd, Zn
Lei 2012	East River basin	Shizhuyuan village, Bailutang village, Matian village, Zitang, Fengyuzhong, Zhangshuxia	0–20	AAS, AFS	Pb, Cd, As, Zn, Hg
Lei 2008	Shizhuyuan mine	Shizhuyuan village	0–20	AAS, ICP-OES, AFS	Pb, Cd, As, Cu, Zn
Lei 2010	Shizhuyuan mine	Shizhuyuan village	0–10	AAS	Pb, Cd, Cu, Zn
Liao 2005	Shizhuyuan village	-	15	HG-AFS	As
Zhou 2016	Shizhuyuan mine	-	Not specified		Pb, Cd, As, Cu, Zn
Zhou 2013	Shizhuyuan mine	-	0–20	AAS	Pb, Cd, Zn
He et al. 2013	East River basin	Zhudui village, Matian village	0–20	AAS	Pb, Cd

ICP-MS = inductively coupled plasma mass spectrometry, SDDC = silver diethyldithiocarbamate colorimetric method, AAS = atomic absorption spectrometry, AFS = atomic fluorescence spectrometry, ICP-OES = inductively coupled plasma optical emission spectroscopy, and HG-AFS = hydride generation atomic fluorescence spectroscopy.

**Table 2:** Heavy metal element pollution risk screening value and regulatory value for non-field agricultural soil.

Heavy Metal *	Risk Screening Values (mg/kg)		Risk Control Values (mg/kg)	
	5.5 < pH ≤ 6.5	6.5 < pH ≤ 7.5	5.5 < pH ≤ 6.5	6.5 < pH ≤ 7.5
Lead (Pb)	90	120	500	700
Cadmium (Cd)	0.3	0.3	2.0	3.0
Arsenic (As)	40	30	150	120
Zinc (Zn)	200	250	-	-
Copper (Cr)	50	100	-	-

* Pb is under 50 mg/kg, Cd is under 0.30 mg/kg, As is under 25 mg/kg, Cu is under 200 mg/kg, Zn in under 250 mg/kg, and Cr is under 250 mg/kg.

**Table 3:** Heavy metal concentrations in agricultural soils collected from different villages of the study area (mg/kg).

Village	Samples (No)	Mean Level of Heavy Metals (Range)				
		Lead	Cadmium	Arsenic	Zinc	Copper
Shizhuyuan	39	961.45 (107.98–4280.61)	5.90 (0.46–11.9)	383.38 (53.96–1217.2)	438.27 (196.1–1064.21)	109.24 (40.43–221.40)
Banqiao	1	1076.08	6.04	-	271.04	-
	6	1088.30 (852.12–1443.73)	7.75 (3.5–11.07)	709.29 (379.34–1226.52)	1000.71 (529.60–1251.59)	135.83 (110.08–148.95)
Jintian	4	321.11 (154.47–658.08)	2.70 (2.25–3.08)	192.49 (144.72–251.02)	416.61 (295.87–512.09)	72.18 (53.64–88.18)
Xiangshanping	1	349.24	5.06	-	196.06	-
Matian	23	352.08 (87.31–1119.70)	2.48 (0.47–7.18)	65.88 (23.82–186.20)	47.81 (9.79–375.11)	4.47 (1.93–11.54)
	135	563.37	11.15	596.31	315.73	73.46
Bailutang	3	267.22 (189.2–423.44)	1.22 (1.10–1.38)	70.29 (55.48–92.45)	468.41 (265.23–616.20)	62.6
Shihupu	82	548.95 (442.25–655.65)	8.13 (6.93–9.32)	351.18 (188.96–513.40)	577.70 (225.73–929.66)	53.75
	64	694.30 (78.50–1234.99)	5.89 (0.92–11.75)	586.93 (47.30–1408.90)	381.27 (270.04–584.47)	51.61 (34.80–70.33)
	87	678.77	10.06	881.38	301.27	77.53
	3	143.77 (66.45–278.86)	0.27 (0.12–0.4)	41.04 (31.15–56.84)	187.35 (112.71–272.36)	-
Zhangshuxia	4	423.02 (70.77–1471.19)	3.59 (0.4–12.76)	190.13 (4.88–709.14)	468.14 (123.26–1465.49)	-
Zhudui	3	131.03 (105.48–161.16)	1.50 (1.30–1.82)	-	11.27 (8.18–16.61)	2.13 (2.04–2.18)
Mean	455	542.76	5.12	369.85	362.97	65.48
Standard deviation		321.20	3.35	288.07	244.96	39.16

**Table 4:** Heavy metal pollution indices for soil samples at the study site.

Site	P_Pb_	P_Cd_	P_As_	P_Zn_	P_Cu_	Ps	Pollution Degree *
Shizhuyuan	8.01	19.67	12.78	1.75	1.09	14.36	Severe pollution
Banqiao	8.97	20.13	-	1.08	-	-	Severe pollution
Guanyin qiao	9.07	25.83	23.64	4.00	1.36	18.99	Severe pollution
Jintian	2.68	9.00	6.42	1.67	0.72	6.61	Severe pollution
Xiangshanping	2.91	16.87	-	0.78	-	-	Severe pollution
Hujiaao	2.93	8.27	2.20	0.19	0.04	5.98	Severe pollution
Matian	4.69	37.17	19.88	1.26	0.73	26.78	Severe pollution
Bailutang	2.23	4.07	2.34	1.87	0.63	3.05	Severe pollution
Zitang	4.57	27.10	11.71	2.31	0.54	19.53	Severe pollution
Shihupu	5.79	19.63	19.56	1.53	0.52	14.39	Severe pollution
Pingshang	5.66	33.53	29.38	1.21	0.78	24.37	Severe pollution
Fengyichong	1.20	0.90	1.37	0.75	-	-	Moderate pollution
Zhangshuxia	3.53	11.97	6.34	1.87	-	-	Severe pollution
Zhudui	1.09	5.00	-	0.05	0.02	-	Moderate pollution

*: means.

**Table 5:** Geographic distribution of blood lead levels in 78 residents from two villages.

Age Group	Villages *	Distribution of Blood Lead Levels (%)				χ^2^	*p*
		0 µg/dL~	5 µg/dL~	10 µg/dL~	20 µg/dL~		
**≤14 years (n = 25)**							
	Matian (n = 14)	0 (0.0%)	11 (78.6%)	3 (21.4%)	0 (0.0%)	3.16	0.21
	Zhudui (n = 11)	2 (18.2%)	8 (72.7%)	1 (9.1%)	0 (0.0%)		
	Subtotal (n = 25)	2 (8.0%)	19 (76.0%)	4 (16.0%)	0 (0.0%)		
**≥15 years~ (n = 53)**							
	Matian (n = 35)	1 (2.9%)	17 (48.5%)	14 (40.0%)	3 (8.6%)	8.70	0.03
	Zhudui (n = 18)	5 (27.8%)	6 (33.3%)	7 (38.9%)	0 (0.0%)		
	Subtotal (n = 53)						
**Total (n = 78)**							
	Matian (n = 49)	1 (2.0%)	28 (57.1%)	17 (34.7%)	3 (6.1%)	7.91	0.05
	Zhudui (n = 29)	7 (24.1%)	14 (48.3%)	8 (27.6%)	0 (0.0%)		

* Matian village with polluted East River irrigation water, Zhudui village with other irrigation water.

**Table 6:** Health status survey summary of 193 residents in the East River Basin region.

Village *	Chronic Disease Prevalence		χ^2^	*p*
	Number of People with Disease	Prevalence (%)		
**Matian (n = 59)**	23	39.0	3.23	0.09
**Zhudui (n = 134)**	35	26.1		

* Village used different water sources for irrigation.
